# Clinical and radiological diversity in genetically confirmed primary familial brain calcification

**DOI:** 10.1038/s41598-017-11595-1

**Published:** 2017-09-21

**Authors:** Shingo Koyama, Hidenori Sato, Ryota Kobayashi, Shinobu Kawakatsu, Masayuki Kurimura, Manabu Wada, Toru Kawanami, Takeo Kato

**Affiliations:** 1 Department of Neurology, Hematology, Metabolism, Endocrinology, and Diabetology, Yamagata University Faculty of Medicine, 2-2-2 Iida-nishi, Yamagata, 990-9585 Japan; 2 Genomic Information Analysis Unit, Department of Genomic Cohort Research, Yamagata University Faculty of Medicine, 2-2-2 Iida-nishi, Yamagata, 990-9585 Japan; 3 Department of Psychiatry, Yamagata University Faculty of Medicine, 2-2-2 Iida-nishi, Yamagata, 990-9585 Japan; 4 Department of Neuropsychiatry, Aizu Medical Center, Fukushima Medical University, 21-2 Maeda, Tanisawa, Kawahigashi, Aizuwakamatsu, Fukushima, 969-3492 Japan; 5 Department of Neurology, Okitama Public General Hospital, 2000 Nishi-otsuka, Kawanishi-machi, Higashi-okitama-gun, Yamagata, 992-0601 Japan

## Abstract

Primary familial brain calcification (PFBC) is a rare neuropsychiatric disorder with characteristic symmetrical brain calcifications. Patients with PFBC may have a variety of symptoms, although they also may be clinically asymptomatic. Parkinsonism is one of the most common movement disorders; however, the underlying mechanism remains unclear. This condition is typically transmitted in an autosomal dominant fashion. To date, mutations in *SLC20A2*, *PDGFRB*, *PDGFB*, and *XPR1* have been reported to cause PFBC. The aim of the study was to identify the genetic cause of brain calcification in probands from three PFBC families and in 8 sporadic patients and to perform clinical and radiological assessments focusing on parkinsonism in mutation carriers. Three familial PFBC probands and their relatives and eight sporadic patients affected with brain calcifications were enrolled in this study. Whole-exome sequencing identified three novel mutations: c.269G > T, p.(Gly90Val) and c.516+1G > A in *SLC20A2* in familial cases, and c.602-1G > T in *PDGFB* in a sporadic patient. The c.516+1G > A mutation resulted in exon 4 skipping in *SLC20A2* (p.Val144Glyfs*85). Dopamine transporter single photon emission computed tomography using ^123^I-ioflupane and ^123^I-metaiodobenzylguanidine cardiac scintigraphy revealed pre-synaptic dopaminergic deficit and cardiac sympathetic nerve dysfunction in two *SLC20A2*-related PFBC patients with parkinsonism.

## Introduction

Primary familial brain calcification (PFBC), also known as Fahr’s disease or idiopathic basal ganglia calcification, is a rare neuropsychiatric disorder^1^. In this disease, most symmetrical calcification occurs in the basal ganglia and other brain regions, including the dentate nuclei, thalami, brainstem, supratentorial white matter, and cerebral cortex, in particular within the occipital lobe. Clinical presentation is characterized by psychiatric signs, cognitive impairment, and movement disorders including chorea, dystonia, athetosis, and parkinsonism. Cerebellar and pyramidal signs, seizures, and headache are also associated with this condition. However, individuals with brain calcification can be clinically asymptomatic^2–4^. This condition is typically transmitted in an autosomal dominant fashion and is genetically heterogeneous^5^. To date, mutations in *SLC20A2*, *PDGFRB*, *PDGFB*, and *XPR1* have been reported to be responsible for PFBC and have been detected in both familial and sporadic cases^6–9^. Nevertheless, these four disease-causing genes do not account for all cases of PFBC, indicating additional genetic heterogeneity. Although true sporadic cases resulting from *de novo* mutations have been reported^10,11^, the majority of seemingly sporadic presentations are thought to be due to an inadequate analysis of asymptomatic family members^2,12^. Whole-exome sequencing (WES) is becoming widely adopted as an efficient strategy to identify disease-causing mutations in genetically heterogeneous diseases. We report here three novel mutations in *SLC20A2* and *PDGFB* responsible for PFBC that were successfully detected using WES. Parkinsonism is one of the most common PFBC-related movement disorders. Although pre-synaptic nigrostriatal dopaminergic dysfunction seems to be involved in the mechanism of PFBC-related parkinsonism^13–16^, the relationship between PFBC and Lewy body pathology remains controversial^17–19^. To elucidate the underlying mechanism of PFBC-related parkinsonism, we performed radiological assessments focusing on parkinsonism. In addition to dopamine transporter single photon emission computed tomography (SPECT) using ^123^I-ioflupane, we examined ^123^I-metaiodobenzylguanidine (MIBG) scintigraphy that is useful to differentiate Lewy body-related disorders from other neurodegenerative disorders in genetically confirmed PFBC patients with parkinsonism^20–23^.

## Results

In this study, we identified three novel mutations responsible for PFBC in two familial cases and one sporadic case. Clinical features and genetic findings of genetically confirmed PFBC patients are summarized in Table 1. On the other hand, we could not identify the causative mutation in the remaining subjects (one familial and seven sporadic cases). Clinical and radiological data of the patients who were negatively screened for the candidate genes are summarized in Supplementary Table 1. The mutation detection strategy is summarized in Supplementary Table 2 (Materials and Methods).

**Table 1:** Clinical and genetic features of genetically confirmed primary familial brain calcification patients in this study.

	Family 1			Family 2		sporadic case 1
	I:2	II:2	III:2	I:1	II:1	
sex/age	F/89	M/62	M/27	M/79	M/52	F/50
symptom	parkinsonism	parkinsonism	asymptomatic	dementia/parkinsonism	depression	depression
MIBG scinti	NE	decreased	NE	decreased	normal	NE
DAT SPECT	NE	decreased	NE	decreased	normal	NE
causative gene	*SLC20A2*			*SLC20A2*		*PDGFB*
mutation	c.516+1G > A, r.431_516del, p.Val144Glyfs*85			c.269G > T, p.(Gly90Val)		c.602-1G > T, p.?
SIFT	not applicable			probably damaging		not applicable
PolyPhen-2	not applicable			deleterious		not applicable
CADD phred-like score	21.7			23.6		19.9
ACMG-AMP recommendation	class 5			class 5		class 5
Human Splicing Finder	not applicable			not applicable		affecting splicing
Exome Variant Server	absent			absent		absent
1000 Genomes Project	absent			absent		absent
ExAC	absent			absent		absent
Human Genetic Variation	absent			absent		absent
dbSNP 138	absent			absent		absent
in-house control	absent			absent		absent

F: female; M: male; NE: not examined; MIBG scinti: ^123^I-metaiodobenzylguanidine scintigraphy; DAT SPECT: dopamine transporter single photon emission computed tomography using ^123^I-ioflupane; SIFT: Sorting Intolerant from Tolerant; CADD: Combined Annotation Dependent Depletion; ACMG-AMP: the American College of Medical Genetics and Genomics and the Association for Molecular Pathology; ExAC: The Exome Aggregation Consortium.

### Case descriptions

#### Family 1

**Figure 1:**
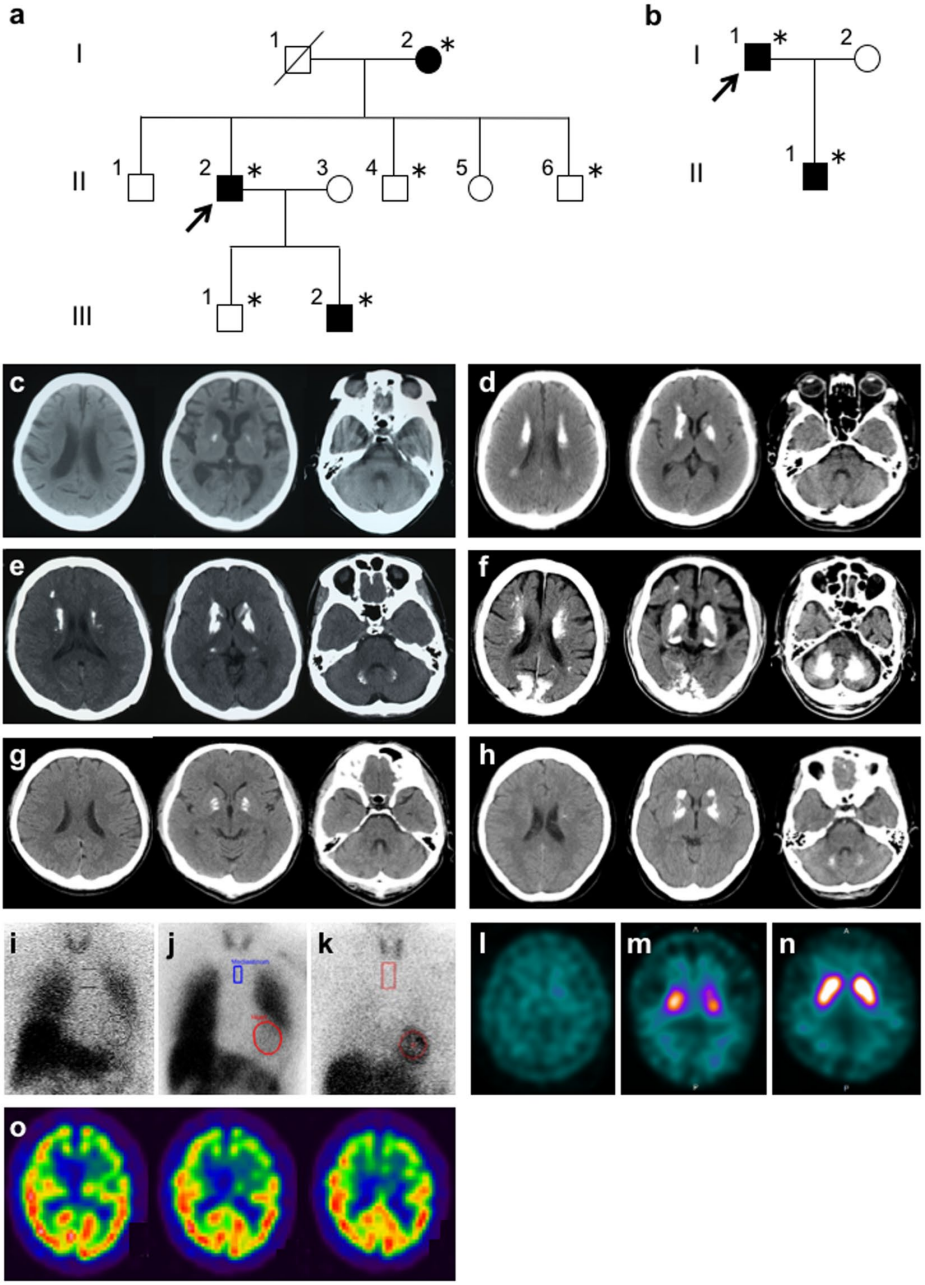
Pedigrees of Family 1 (**a**) and Family 2 (**b**). *Squares*: males; *circles*: females. The arrowheads denote the probands. The filled symbols represent the subjects with calcinosis. Asterisks represent individuals who are included in this study. Brain computed tomography (CT) images demonstrating brain calcinosis in patients of this study with genetically confirmed primary familial brain calcification (**c–h**). (**c**) the proband’s mother of Family 1; (**d**) the proband of Family 1; (**e**) the proband’s affected son; (**f**) the proband of Family 2; (**g**) the proband’s son of Family 2; (**h**) sporadic case 1. ^123^I-metaiodobenzylguanidine scintigraphy in the proband of Family 1 (**i**), the proband of Family 2 (**j**), and the proband’s son of Family 2 (**k**). Dopamine transporter single photon emission computed tomography (SPECT) using ^123^I-ioflupane (**l–n**) in the proband of Family 1 (**l**) and the proband of Family 2 (**m**) showing a decrease of striatal tracer uptake. In the proband’s son of Family 2, tracer uptake is normal (**n**). Brain 99mTc-ethyl cysteinate dimer SPECT in the proband of Family 2 (**o**) showing a hypoperfusion in the frontotemporal lobes with a left-side predominance.

The proband of Family 1 (II:2; Fig. 1a) was a 62-year-old Japanese man who presented with a 2-year history of slowness and gait disturbance. Neurological examination revealed resting tremor in the left hand, bradykinesia, and small shuffling gait. There were no pathological reflexes or signs of ataxia. The Mini-Mental State Examination (MMSE) score was 30/30. His parkinsonism responded to treatment with L-dopa/carbidopa. Brain computed tomography (CT) showed calcification in the lenticular nuclei, caudate nuclei, and deep white matter (Fig. 1d). Decreased cardiac uptake in ^123^I- MIBG scintigraphy was observed (the early image heart/mediastinum ratio was 1.43) (Fig. 1i). Dopamine transporter SPECT using ^123^I-ioflupane demonstrated a marked decrease of striatal tracer uptake with a right-side predominance (Fig. 1l). The 89-year-old mother of the proband (I:2; Fig. 1a) was in a bedridden state with resting tremor in her left hand. Her brain CT revealed a mild calcification in the bilateral lenticular nuclei (Fig. 1c). Considering her age, calcified lesions could be considered as physiological. She was not treated with anti-parkinsonian drugs. The 27-year-old son of the proband (III:2; Fig. 1a) was clinically asymptomatic. His brain CT showed calcification in the lenticular nuclei, caudate nuclei, thalami, subcortical white matter, and dentate nuclei (Fig. 1e). No calcified lesions were detected in the proband’s brothers (II:4 and II:6; Fig. 1a) and the proband’s son (III:1; Fig. 1a) on brain CT images. Calcified lesions on brain CT were observed across three generations in this family. In addition, anticipation of CT imaging was noticed, although the affected proband’s son (III:2; Fig. 1a) had not exhibited any neuropsychiatric symptoms.

#### Family 2

The proband of Family 2 (I:1; Fig. 1b) was a 79-year-old Japanese man presenting with a 5-year history of dementia. His MMSE score was 13/30. His psychiatric symptoms were decreased motivation, irritability, shouting, disinhibition, and impulsive violent behavior. Impaired attention was evident on the digit span task (four digits forward and three digits backward). The number of words recalled on 10-word recall subtest of Alzheimer's Disease Assessment Scale was 1/10. His Frontal Assessment Battery score was 3 of 18 with deficits in all of the items except environmental autonomy. Neither visual nor auditory hallucinations were evident. Fluctuations in cognitive function were not apparent. The patient also exhibited mild parkinsonism with a right-side predominance. His brain CT revealed marked calcified lesions in the lenticular nuclei, caudate nuclei, thalami, dentate nuclei, subcortical white matter, and occipital cortical-subcortical regions (Fig. 1f). Decreased cardiac uptake in ^123^I-MIBG scintigraphy was demonstrated (the early image heart/mediastinum ratio was 1.62) (Fig. 1j). A left-side predominant decrease of tracer uptake in the striatum was observed in dopamine transporter SPECT (Fig. 1m). Brain 99mTc-ethyl cysteinate dimer (99mTc-ECD) SPECT revealed left-side predominant frontotemporal hypoperfusion (Fig. 1o). The 52-year-old son of the proband was diagnosed with depression at the age of 50 years. He also experienced headache. Neurological examination revealed no signs of ataxia or parkinsonism. His MMSE score was 30/30. His brain CT showed calcification in the lenticular nuclei (Fig. 1g). Cardiac uptake of ^123^I-MIBG was normal (the early image heart/mediastinum ratio was 3.24) (Fig. 1k). Dopamine transporter SPECT demonstrated a normal uptake in the striatum (Fig. 1n).

#### Sporadic case 1

A 50-year-old woman referred besecause of ptosis and diplopia was diagnosed with myasthenia gravis, based on a positive response to intravenous edrophonium chloride and the presence of antibodies to acetylcholine receptor. She was successfully treated with ambenonium chloride and tacrolimus. Brain CT performed to assess her ocular manifestations incidentally disclosed calcinosis in the lenticular nuclei, caudate nuclei, and dentate nuclei (Fig. 1h). She was also diagnosed as being in a depressive state. There were no other neurological symptoms other than those related to myasthenia gravis. She had no family history of any neuropsychiatric disorders.

### Genetics

**Figure 2:**
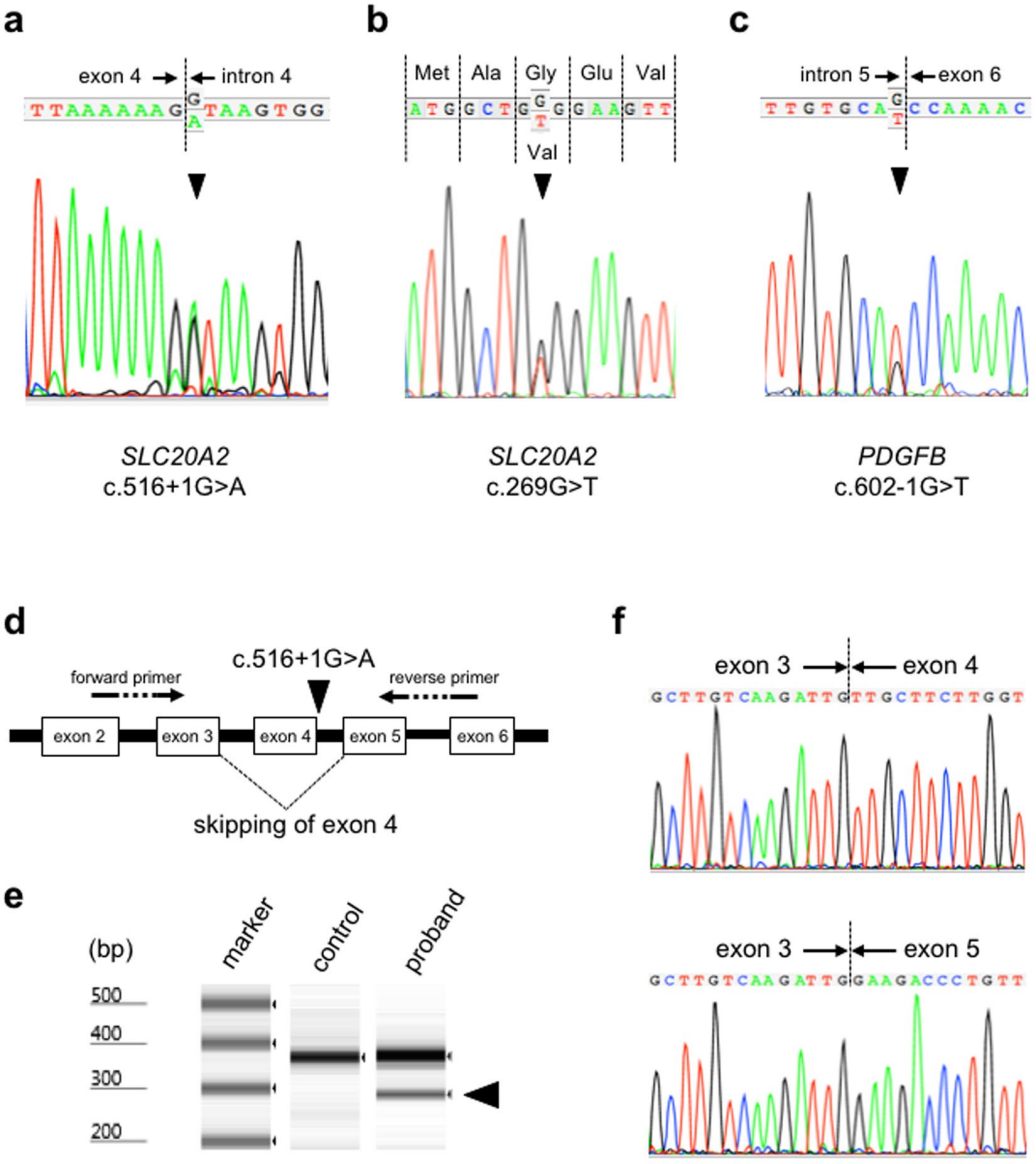
Electropherograms of Sanger sequences of the proband of Family 1 (**a**), the proband of Family 2 (**b**), and sporadic case 1 (**c**). Arrowhead in A indicates the c.516+1G > A mutation in *SLC20A2*. Arrowhead in B shows the c.269G > T mutation in *SLC20A2*. Arrowhead in C represents the c.602-1G > T mutation in *PDGFB*. (**d**) Schematic representation of the c.516+1G > A mutation in *SLC20A2* and the primers used in reverse transcription-polymerase chain reaction (RT-PCR) analysis to demonstrate the skipping of exon 4. (**e**) RT-PCR analysis showing another transcript in the proband of Family 1 carrying the c.516+1G > A mutation in *SLC20A2*. In addition to a PCR product of the expected size (355 bp), a fast-migrating band is observed (arrowhead). (**f**) Sanger sequencing of the RT-PCR products demonstrating that the c.516+1G > A mutation in *SLC20A2* leads to the skipping of exon 4.

PFBC is a clinically and genetically heterogeneous condition. Therefore, amplicon-based next-generation sequencing of the three familial probands and the eight sporadic patients was performed to determine the causative mutations in this study. We performed genetic analysis according to the mutation detection strategy (Supplementary Table 2, Materials and Methods). The mean read depth and the total number of detected variants by the two pipelines constructed with Bowtie2-GATK (the Genome Analysis Toolkit) and BWA (Burrows Wheeler Alignment)-Platypus at the whole exome level are summarized in Supplementary Table 3. Coverage for the four genes are summarized in Supplementary Table 4. The number of detected variants called by the two pipelines and the disease-causing variants obtained after filtering process are also shown in Supplementary Table 4. According to the filtering process (Supplementary Table 2), we identified three novel mutations in genes known to be responsible for PFBC: two of them were detected in *SLC20A2*. A G-to-A transition at the +1 splice donor position in intron 4 of *SLC20A2* (c.516+1G > A) was revealed in the proband of Family 1 in a heterozygous state (Fig. 2a). Human Splicing Finder (HSF) predicted this mutation to affect splicing through alteration of the wild-type donor site. Sanger sequencing confirmed that only subjects with brain calcifications on CT carried the mutation in this family. Reverse transcription-polymerase chain reaction (RT-PCR) analysis of the patient showed an additional fast-migrating band in addition to a PCR product of the expected size (Fig. 2 d, e). Sanger sequencing demonstrated that the mutation resulted in the skipping of exon 4 of *SLC20A2* (r.431_516del, p.Val144Glyfs*85) (Fig. 2f). In the proband of Family 2, WES detected a heterozygous G-to-T transversion in the coding region of exon 2 (c.269G > T), resulting in a glycine-to-valine substitution at position 90, p.(Gly90Val) in *SLC20A2* (Fig. 2b). Sanger sequencing identified the c.269G > T mutation in both the proband and his affected son. This mutation was predicted as probably damaging and deleterious by Polyphen-2 and Sorting Intolerant from Tolerant (SIFT), respectively. In sporadic case 1, we found a heterozygous G-to-T transversion in *PDGFB* located at the base pair immediately flanking the 5′ end of exon 6 (c.602-1G > T, p.?) (Fig. 2c). This variant was predicted to lead to the alteration of the wild-type acceptor site by HSF. The three detected variants were considered to be deleterious by Combined Annotation Dependent Depletion (CADD) (all phred-like scores were higher than 15, Table 1). The above two splicing variants were classified as pathogenic (class 5) and the missense *SLC20A2* variant was considered as likely pathogenic (class 4) according to the recommendation of the American College of Medical Genetics and Genomics and the Association for Molecular Pathology (ACMG-AMP) (Table 1). The above mutations were not detected in publicly available databases: NHLBI Exome Sequencing Project (ESP6500), dbSNP 138, 1000 Genomes Project, The Exome Aggregation Consortium (ExAC), Human Genetic Variation Database (HGVD), and in-house exome sequencing data of 128 control subjects. Furthermore, these mutations were not listed in the Human Gene Mutation Database (HGMD) or ClinVar. In additon, we assessed copy number variations (CNVs) using WES data, but we could not detect CNVs in the candidate four genes responsible for PFBC.

## Discussion

*SLC20A2* mutations are a major cause of PFBC and account for about one-half of all familial cases. To date, more than 40 disease-causing mutations have been identified^2,24^. *SLC20A2* [solute carrier family 20 (phosphate transporter) member 2] encodes the type III sodium-dependent inorganic phosphate (Pi) transporter 2, PiT-2. Mutagenesis analysis in *Xenopus laevis* oocytes showed that disease-causing mutants decreased Pi uptake activity without an obvious effect on the wild-type PiT-2 function, indicating that the underlying mechanism is due to haploinsufficiency rather than a dominant-negative effect^6^. This idea is supported by a PFBC case with a heterozygous deletion of the entire *SLC20A2* gene^25^. In addition, partial, mono- or di-exonic *SLC20A2* deletions have also been reported to cause this condition^26^. Jensen *et al*. reported that *Slc20a2* knockout mice demonstrate brain calcifications, suggesting that the functional deficit of PiT-2 is implicated in the pathophysiology of *SLC20A2*-related PFBC^27^. In our study, WES of the proband of Family 1 successfully detected a novel splice donor site mutation, c.516+1G > A. Sanger sequencing of the family members revealed that the mutation completely co-segregated with the calcification phenotype within the pedigree. RT-PCR analysis demonstrated that the mutation led to a frameshift and a premature stop codon. The truncated protein is considered to be functionally null. In addition, we found a novel missense mutation, p.(Gly90Val), in Family 2. Glycine90 is located in the third transmembrane domain of PiT-2 and is present within the ProDom domain, characteristic for all PiT family members. Although the exact effect of this mutation on PiT-2 function is unclear, the residue is evolutionarily conserved from *Homo sapiens* to *Danio rerio*, supporting its functional importance.

Mutations in *PDGFB*, the gene encoding platelet derived growth factor β (PDGF-B), the main ligand for platelet-derived growth factor receptor β (PDGF-Rβ), have been reported to cause PFBC^8^. *PDGFB* is thought to be the second most common causative gene after *SLC20A2* for this condition^28,29^. To date, 13 mutations in *PDGFB* have been found^8,10,29–33^, including three nonsense mutations (p.Gln145*, p.Gln147*, and p.Arg149*) and five missense mutations (p.Leu9Arg, p.Ala95Thr, p.Leu110Val, p.Leu119Pro, and p.Pro122Leu). A splice-site mutation, c.64-3C > G, is predicted to result in a premature stop codon. The c.3G > A and c.3G > C mutations replace the start methionine and c.726G > C (p.*242Tyrext*89) substitutes the stop codon with a tyrosine, resulting in an extended PDGF-B protein. PFBC-associated PDGF-B mutations are thought to cause complete loss of function through either reduced protein levels of PDGF-B or abolished PDGF-Rβ phosphorylation^34^. In addition, a partial intragenic large deletion within *PDGFB*, c.161-238_602-676del441 (p.Glu54Aladel147) has been reported in a patient with brain calcification and leukoencephalopathy, indicating a loss-of-function mechanism for *PDGFB*-related PFBC^30^. This hypothesis is supported by the detection of brain calcifications in PDGF-B retention motif knockout mice^8^. Furthermore, mutations in *PDGFRB*, which encodes for the transmembrane receptor PDGF-Rβ are also responsible for PFBC^6^. Five missense mutations (p.Leu658Pro, p.Arg695Cys, p.Asp737Asn, p.Arg987Trp, and p.Glu1071Val) and the c.3G > A variant have been reported^3,7,35,36^. Sanchez-Contreras *et al*. reported that the PFBC-causing p.Arg695Cys and p.Leu658Pro mutations cause impairment of PDGF-Rβ autophosphorylation in a cell culture with PDGF-BB, a recombinant human PDGF-B homodimer. The p.Arg987Trp mutation resulted in a decreased level of PDGF-Rβ protein because of accelerated degradation by the proteasome^35^. Taken together, an impairment of the PDGF-B/PDGF-Rβ pathway could cause brain calcification. Interestingly, mutations in *PDGFRB* also cause autosomal-dominant infantile myofibromatosis, Penttinen syndrome, and a novel overgrowth syndrome characterized by somatic overgrowth, distinctive facial features, hyper-elastic and fragile skin, white matter lesions, and neurologic deterioration^37–40^. In contrast to the PFBC-related mutations, the Penttinen syndrome-causing p.Val665Ala PDGF-Rβ variant exhibits robust ligand-independent autophosphorylation and constitutive PDGF-Rβ signaling through STAT3 and PLCγ^38^. The p.Arg561Cys mutation in familial infantile myofibromatosis is predicted *in silico* to decrease autoinhibition of PDGF-Rβ and lead to high PDGF-Rβ signaling activity, suggesting that a gain-of-function mechanism contributes to the pathogenesis of this condition^39^. In this study, we identified a novel splice acceptor site mutation in *PDGFB*. Although we have not examined the exact effect on splicing at the transcript level, the c.602-1G > T mutation is predicted *in silico* to affect splicing, supporting the idea that loss-of-function mutations in *PDGFB* cause PFBC.

In the present study, we identified three novel mutations in *SLC20A2* and *PDGFB*. On the other hand, we could not detect the causative mutation in the known PFBC-causing genes in the remaining cases. Mutations in *SLC20A2*, *PDGFRB*, *PDGFB*, and *XPR1* have been reported to be responsible for PFBC^6–9^; however, these four disease-causing genes do not account for all cases of PFBC, indicating additional genetic heterogeneity^5^. One mutation was detected in a seemingly sporadic case in this study. Some patients with a sporadic presentation have been diagnosed with definite PFBC based on genetic analysis. Strictly speaking, the absence of a family history of PFBC-associated signs in relatives is not sufficient to exclude inheritance because of its incomplete clinical penetrance, whereas true sporadic cases due to *de novo* mutations have been reported^10,11^. Therefore, we should be careful to ensure that patients with PFBC are really sporadic without genetic analysis and/or brain imaging of their first-degree relatives. PFBC patients exhibit a variety of clinical presentations. Furthermore, clinical diversity is recognized within and between families with PFBC^3^. In Family 2, the proband had dementia and parkinsonism, whereas his son was diagnosed with depression but not with dementia or parkinsonism at the time of diagnosis. On the other hand, patients with the p.Ser637Arg *SLC20A2* mutation exhibit similar neurological symptoms^17^. In the present study, interestingly, the proband of Family 1 and his mother also had a similar clinical presentation of asymmetric parkinsonism including bradykinesia and resting tremor.

Parkinsonism is the most common symptom after dystonia in PFBC-related movement disorders^4^. As observed in the present cases, pre-synaptic nigrostriatal dopaminergic dysfunction was reported in patients with PFBC^13–16^. Nevertheless, although the exact reason is unclear, the response to L-dopa therapy is different among patients. Saito *et al*. showed that the post-synaptic dopaminergic impairment was also observed in a case of PFBC^14^. The degree of post-synaptic dopaminergic disability in the striatum might explain variations in the effectiveness of anti-parkinsonian medications. The relationship between PFBC and Lewy body pathology remains controversial. Manyam *et al*. reported a familial case of PFBC, in which the proband presenting with parkinsonism showed Lewy bodies indicative of Parkinson’s disease in the substantia nigra, while another patient from the same family with clinical evidence of parkinsonism did not demonstrate Lewy body pathology^18^. As previously mentioned, therefore, it is possible that the co-occurrence of PFBC and Lewy body-related conditions is incidental^17,18^. However, neuropathological examinations revealed neuronal loss and Lewy bodies in *SLC20A2*-related PFBC patients presenting with parkinsonism^17,19^. Further genetic and neuropathological examinations of PFBC are required to answer the question of whether Lewy bodies are age-related incidental findings or whether they have certain associations with the pathomechanisms underlying PFBC. Interestingly, although pathological examinations were not performed in the present study, in addition to the decrease of tracer uptake in dopamine transporter SPECT, decreased cardiac uptake in ^123^I-MIBG scintigraphy was also demonstrated in our patients with parkinsonism, consistent with Lewy body-related conditions such as Parkinson’s disease and dementia with Lewy bodies^23^. In fact, in the proband of Family 1, his parkinsonism was clinically indistinguishable from idiopathic Parkinson’s disease. A reduction in cardiac MIBG uptake was also revealed in the proband of Family 2; however, the clinical picture, including brain 99mTc-ECD SPECT findings, was similar to that of frontotemporal lobar degeneration rather than dementia with Lewy bodies, except that parkinsonism was also present.

In conclusion, we identified three novel mutations in *SLC20A2* and *PDGFB* responsible for PFBC in two familial cases and one sporadic case using WES. Genetic analysis should be considered, even in apparently sporadic cases, for the precise diagnosis of PFBC because clinical penetrance is incomplete. To elucidate the underlying mechanism of PFBC-related parkinsonism, radiological assessments including ^123^I-MIBG cardiac scintigraphy and dopamine transporter SPECT in a larger sample of patients are required.

## Materials and Methods

### Subjects

Seven patients and five relatives from three PFBC families with an autosomal dominant inheritance and eight sporadic cases were enrolled in this study. The clinical criteria for PFBC are described elsewhere^41^. The serum levels of calcium, phosphorus, and parathyroid hormone were all within normal limits in the patients. Subjects with brain calcifications on CT were classified as affected regardless of their clinical status. Genomic DNA was extracted from the peripheral blood of the patients and relatives after obtaining their written informed consent. This study was approved by the Medical Ethics Committee of the Yamagata University Faculty of Medicine. All experiments were performed in accordance with the Declaration of Helsinki.

### Mutation detection strategy

To determine the causative mutations, we performed genetic analysis according to the mutation detection strategy in this study (Supplementary Table 2).

### Whole exome sequencing

WES of the three familial probands and of eight sporadic patients was performed using amplicon-based, next-generation sequencing as previously described^42^. Briefly, libraries were constructed using an Ion AmpliSeq Library Kit v2.0 (Life Technologies, Carlsbad, CA, USA) according to the manufacturer’s instructions. Quantification of the libraries was performed on a 2200 TapeStation Instrument using High Sensitivity D1000 Reagents and High Sensitivity D1000 ScreenTape (Agilent Technologies, Santa Clara, CA, USA). Amplified libraries were submitted to emulsion PCR using an Ion OneTouch™ 2 Instrument with an Ion PI™ Template OT2 200 Kit v3. Ion sphere particles were enriched using Ion OneTouch ES and were loaded on an Ion PI Chip v2. Sequencing was performed by Ion Proton™ with an Ion PI Sequencing 200 Kit v3. Read sequence files were run through two independent pipelines constructed with Bowtie2 (http://bowtie-bio.sourceforge.net/bowtie2/index.shtml)-GATK (https://www.broadinstitute.org/gatk/index.php) and BWA (http://bio-bwa.sourceforge.net/bwa.shtml)-Platypus (http://www.well.ox.ac.uk/platypus) to obtain variant call format files. Then, concordant genetic variants detected by the two pipelines were annotated using ANNOVAR (http://annovar.openbioinformatics.org). We focused on genes previously reported to be responsible for PFBC (*SLC20A2*, *PDGFB*, *PDGFRB*, and *XPR1*) and picked up the variants in the coding regions and exon-intron boundaries of these four genes.

### Variant analysis

To avoid missing variants, BAM files were loaded into Integrative Genomics Viewer (IGV; http://www.broadinstitute.org/igv) for visual inspection for the candidate four genes. After excluding synonymous mutations, the variants with a minor allele frequency of less than 0.1% in the following databases were extracted: ESP6500 (http://evs.gs.washington.edu), dbSNP 138 (http://www.ncbi.nlm.nih.gov), 1000 Genomes Project (http://www.1000genomes.org), ExAC (http://exac.broadinstitute.org), and HGVD (http://www.genome.med.kyoto-u.ac.jp/SnpDB/). The detected variants were queried in the two mutation databases: HGMD (http://www.hgmd.org) and ClinVar (https://www.ncbi.nlm.nih.gov/clinvar/). The pathological potential of the identified variants was evaluated using *in silico* prediction methods: PolyPhen-2 (http://genetics.bwh.harvard.edu/pph2/) and SIFT (http://sift.bii.a-star.edu.sg/) for the missense variant, HSF (http://www.umd.be/HSF3/HSF.html) for the splice-site variants, and CADD (http://cadd.gs.washington.edu/info) for both splice site and nonsynonymous variants. We followed the ACMG-AMP recommendation for the clinical interpretation of the detected variants^43^. To detect CNVs using WES data, we ran a hidden Markov model algorithm as previously described^42^. The nomenclature of the detected variants refers to transcripts NM_006749.4 for *SLC20A2* and NM_002608.3 for *PDGFB*.

### Sanger sequencing

Regarding the target regions covered by five or fewer reads, Sanger sequencing was performed to examine all the coding regions and exon-intron boundaries of the four genes. In addition, the mutations identified by WES were confirmed by Sanger sequencing. Primers sequences are described elsewhere^6–9^. PCR products were sequenced using a BigDyeV3.1 terminator Kit on an ABI 3100 automated sequencer (Life Technologies).

### RT-PCR

Peripheral blood mononuclear cells were isolated using Ficoll-Paque Plus (GE Healthcare, Uppsala, Sweden). Total RNA was purified with the RNeasy Mini Kit (Qiagen, Maryland, USA) according to the manufacturer’s instructions. RNA was treated with DNase (RNase-Free DNase Set; Qiagen) to avoid contamination of genomic DNA. First-strand cDNA was synthesized using Ready-to-Go You Prime First-Strand Beads (GE Healthcare) with random hexamers (Random primer; TaKaRa, Shiga, Japan). To examine the effect of the *SLC20A2* mutation on splicing, RT-PCR was carried out with a forward primer spanning exons 2 and 3 (5′-ttagtgccatggttggttcc-3′) and a reverse primer straddling exons 5 and 6 (5′-gagaacaaggccgagcact-3′). The size of PCR products was assessed using a 2200 TapeStation Instrument. The amplified PCR fragments were subcloned into pGEM-T Easy vector (Promega, Madison, WI, USA) and were sequenced with a T7 promoter primer on an ABI 3100 automated sequencer.

### Data availability

All data generated or analyzed during this study are included in the article.

## Electronic supplementary material

Supplementary Tables
