# Supplementary material for: Clinical and radiological diversity in genetically confirmed primary familial brain calcification
Source: Sci Rep. 2017 Sep 21;7:12046. doi: 10.1038/s41598-017-11595-1 (PMC5608910; doi:10.1038/s41598-017-11595-1)
Supplement: Supplementary file 1 — Supplementary Tables [file 41598_2017_11595_MOESM1_ESM.pdf]

Clinical and radiological diversity in genetically confirmed primary familial brain calcification

Shingo Koyama[1], Hidenori Sato[2], Ryota Kobayashi[3], Shinobu Kawakatsu[4], Masayuki Kurimura[5], Manabu Wada[1], Toru Kawanami[1], and Takeo Kato[1].

[1] Department of Neurology, Hematology, Metabolism, Endocrinology, and Diabetology, Yamagata University Faculty of Medicine, 2-2-2 Iida-nishi, Yamagata 990-9585, Japan.

[2] Genomic Information Analysis Unit, Department of Genomic Cohort Research, Yamagata University Faculty of Medicine, 2-2-2 Iida-nishi, Yamagata 990-9585, Japan.

[3] Department of Psychiatry, Yamagata University Faculty of Medicine, 2-2-2 Iida-nishi, Yamagata 990-9585, Japan.

[4] Department of Neuropsychiatry, Aizu Medical Center, Fukushima Medical University, 21-2 Maeda, Tanisawa, Kawahigashi, Aizuwakamatsu, Fukushima 969-3492, Japan.

[5] Department of Neurology, Okitama Public General Hospital, 2000 Nishi-otsuka, Kawanishi-machi, Higashi-okitama-gun, Yamagata 992-0601, Japan.

Supplementary Table 1. Clinical and radiological data of the negatively screened patients in this study.

	Family 3		sporadic						
	proband	proband's daughter	case 2	case 3	case 4	case 5	case 6	case 7	case 8
sex/age	M/55	F/16	F/76	F/82	F/73	F/88	M/67	F/74	F/76
symptom	depression	asymptomatic	asymptomatic	dementia	asymptomatic	dementia	dementia	dementia	dementia
Calcification on CT	L, D, S	L	L, D, S	L	L	L, D	L, D, S	L, D	L, D, S
MIBG scinti	NE	NE	NE	normal	normal	normal	NE	NE	decreased
DAT SPECT	NE	NE	NE	NE	normal	normal	NE	NE	decreased

CT: computed tomography; L: the lenticular nuclei; D: the dentate nuclei; S: subcortical white matter; MIBG scinti: ^{123}I-metaiodobenzylguanidine scintigraphy; DAT SPECT: dopamine transporter single photon emission computed tomography using ^{123}I-ioflupane.

Supplementary Table 2. Mutation detection strategy in this study.

Mutation detection strategy	
1	Whole exome sequencing
2	Bowtie2-GAKT/BWA-Platypus
3	Annotation using ANNOVAR
4	Visual inspection using IGV for the four genes (*SLC20A2*, *PDGFB*, *PDGFRB*, and *XPR1*)
5	Sanger sequencing (regions of the four genes covered by five or fewer reads)
6	Variants in coding regions and adjacent splice sites in the four genes
7	Exclusion of synonymous variants
8	Population frequency < 0.1% in publicly available databases (ESP6500, dbSNP 138, ExAC, 1000 Genomes Project, and HGVD)
9	Disease databases: HGMD and ClinVar
10	Validation by Sanger sequencing
11	Assessment of the variants by Polyphen-2, SIFT, HSF, and CADD
12	Variant interpretation by the ACMG-AMP recommendations
13	Assessment of copy number variations using whole-exome sequencing data

GATK: the Genome Analysis Toolkit; BWA: Burrows Wheeler Alignment; ICG: Integrative Genomics Viewer; ExAC: The Exome Aggregation Consortium; HGVD: Human Genetic Variation Database; HGMD: the Human Gene Mutation Database; SIFT: Sorting Intolerant from Tolerant; HSF: Human Splicing Finder; CADD: Combined Annotation Dependent Depletion; ACMG-AMP: the American College of Medical Genetics and Genomics and the Association for Molecular Pathology.

Supplementary Table 3. Mean read depth and the total number of detected variants at the whole exome level in the three familial probands and eight sporadic cases.

	Family 1	Family 2	Family 3	sporadic							
				case 1	case 2	case 3	case 4	case 5	case 6	case 7	case 8
Mean read depth [a]	57.01	47.49	25.41	49.91	49.35	38.99	28.85	65.62	24.12	44.81	46.42
Number of variants [b]	22314	27310	24232	22093	19598	18724	21471	23156	22473	22491	20907

[a] The mean read depth obtained by Bowtie2-GAKT (the Genome Analysis Toolkit).

[b] The total number of variants called by two pipelines constructed with Bowtie2-GAKT/BWA (Burrows Wheeler Alignment)-Platypus at the whole exome level.

Supplementary Table 4. Coverage and the number of the variants of four candidate genes in the present study.

		Family 1	Family 2	Family 3	sporadic							
					case 1	case 2	case 3	case 4	case 5	case 6	case 7	case 8
gene	n-fold	coverage (%)										
SLC20A2	5	100	100	100	100	99.9	85.4	93.1	100	100	100	100
	10	100	100	100	100	97	78.4	93.1	100	97.4	100	100
	20	100	100	93.1	100	83.2	65.6	85	97.6	84.6	98.7	100
PDGFB	5	100	100	100	100	100	88.4	100	100	100	100	100
	10	100	100	88.4	100	100	88.4	98.9	100	100	94.7	100
	20	100	100	81.5	100	100	56	64.7	79.0	85.8	77.9	100
PDGFRB	5	83.7	82.6	77.4	83.9	85.3	74.9	76.3	90.0	76.5	83.9	82.8
	10	81.2	81.2	69.8	75.3	83.3	59.0	74.4	84.6	71.4	74.6	77.6
	20	75.6	77.4	65.2	70.2	78.7	35.7	72.9	74.4	66.0	69.1	71.4
XPR1	5	100	100	100	100	99.9	83.2	84.1	96.6	93.2	97.2	100
	10	100	100	99.6	99.3	96.6	73.6	81.7	96.6	82.5	94.2	99.9
	20	96.5	91.4	87.2	90.6	85.2	40.1	74.5	88.3	69.3	84.8	92.3
variants	before [a]	2	4	1	1	4	2	3	3	4	1	0
	after [b]	1	1	0	1	0	0	0	0	0	0	0

[a] The number of variants called by two pipelines constructed with Bowtie2-GAKT (the Genome Analysis Toolkit)/BWA (Burrows Wheeler Alignment)-Platypus in the three familial probands and eight sporadic cases.

[b] The disease-causing variants in the present study obtained after filtering process described in Supplementary Table 2.
